# Effect of vasopressin antagonism on renal handling of sodium and water and central and brachial blood pressure during inhibition of the nitric oxide system in healthy subjects

**DOI:** 10.1186/1471-2369-15-100

**Published:** 2014-06-25

**Authors:** Safa Al Therwani, Frank Holden Mose, Janni Majgaard Jensen, Jesper Nørgaard Bech, Erling Bjerregaard Pedersen

**Affiliations:** 1 University Clinic in Nephrology and Hypertension, Department of Medical Research, Holstebro Hospital and Aarhus University, Hospital Unit Jutland West, Laegaardvej 12, 7500 Holstebro, Denmark

**Keywords:** Tolvaptan, Nitric oxide, AQP2, ENaC, Blood pressure, AVP, Renin, Angiotensin II

## Abstract

**Background:**

Tolvaptan is a selective vasopressin receptor antagonist (V2R) that increases free water excretion. We wanted to test the hypotheses that tolvaptan changes both renal handling of water and sodium and systemic hemodynamics during basal conditions and during nitric oxide (NO)-inhibition with L-NG-monomethyl-arginine (L-NMMA).

**Methods:**

Nineteen healthy subjects were enrolled in a randomized, placebo-controlled, double-blind, crossover study of two examination days. Tolvaptan 15 mg or placebo was given in the morning. L-NMMA was given as a bolus followed by continuous infusion during 60 minutes. We measured urine output(UO), free water clearance (C_H2O_), fractional excretion of sodium (FE_Na_), urinary aquaporin-2 channels (u-AQP2) and epithelial sodium channels (u-ENaC_γ_), plasma vasopressin (p-AVP), central and brachial blood pressure(cBP, bBP).

**Results:**

During baseline conditions, tolvaptan caused a significant increase in UO, C_H2O_ and p-AVP, and FE_Na_ was unchanged. During L-NMMA infusion, UO and C_H2O_ decreased more pronounced after tolvaptan than after placebo (-54 vs.-42% and -34 vs.-9% respectively). U-AQP2 decreased during both treatments, whereas u-ENaC_γ_ decreased after placebo and increased after tolvaptan. CBP and bBP were unchanged.

**Conclusion:**

During baseline conditions, tolvaptan increased renal water excretion. During NO-inhibition, the more pronounced reduction in renal water excretion after tolvaptan indicates that NO promotes water excretion in the principal cells, at least partly, via an AVP-dependent mechanism. The lack of decrease in u-AQP2 by tolvaptan could be explained by a counteracting effect of increased plasma vasopressin. The antagonizing effect of NO-inhibition on u-ENaC suggests that NO interferes with the transport via ENaC by an AVP-dependent mechanism.

## Background

Vasopressin (AVP) stimulates translocation of aquaporin2 water channels (AQP2) to the apical plasma membrane, thereby increasing water permeability in the collecting ducts [1-3]. In addition to its effects on water permeability AVP also stimulate sodium absorption in the collecting ducts by increasing the activity of the eprthelial sodium channels (ENaC) [4,5]. In addition to AVP, recent studies have suggested that nitric oxide (NO) also has an effect on the translocation of AQP2 to form water channels [6-8]. The synthesis of NO is inhibited by L-NG-monomethyl-arginine (L-NMMA), a competitive inhibitor of NO. Infusion of L-NMMA causes reduced renal plasma flow, reduced renal excretion of sodium, renal renin concentration and increased blood pressure [7,9]. However, the effects of NO inhibition on the translocation of AQP2 water channels during inhibition of the vasopressin 2 receptor (V2R) have not been examined.

Tolvaptan is a selective V2R antagonist that inhibits activation of V2R by AVP thereby inhibiting insertion of AQP2 water channels in the collecting ducts, and thus stimulating aquaresis, an electrolyte-free water excretion [2,10-19]. We wanted to test the hypotheses that short-term tolvaptan treatment would decrease renal absorption of water and sodium during basal conditions, and that this effect would be antagonized by NO-inhibition. In addition, we wanted to test whether the effects of tolvaptan on the central blood pressure, vasopressin in plasma and the activity of the renin-angiotensin-aldosterone system antagonized the effects on renal water and sodium handling.

Selctive V2R antagonism may be therapeutically useful for the treatment of conditions with impaired water excretion, and consequently dilutional hyponatremia, associated with the syndrome of inappropriate secretion of antidiuretic hormone, congestive heart failure and cirrhosis. Increased knowledge of the mechanisms involved in the regulation of water and sodium may improve future treatment of hyponatremia in these patients.

We conducted a randomized, placebo-controlled, double-blinded, crossover study in healthy subjects to measure the effects of tolvaptan on (1) renal handling of water and sodium (glomerular filtration rate (GFR), urine output (UO), free water clearance (C_H2O_), u-AQP2, u-EnaCγ, fractional excretion of sodium (FE_Na_), (2) plasma concentrations of renin (PRC), angiotensin II (p-AngII), aldosterone (p-Aldo) and p-AVP, (3) hemodynamics (brachial blood pressure (bBP), central blood pressure (cBP), pulse wave velocity (PWV) and augmentation index (AI)), during baseline conditions and during and after inhibition of the nitric oxide synthesis.

## Methods

### Subjects

#### *Inclusion criteria*

Healthy subjects were enrolled with the inclusion criteria 1) age between 18–40 yrs., 2) men and women, 3) non-smokers, 4) BMI between 18.5 and 30 kg/m^2^.

#### *Exclusion criteria*

Exclusion criteria included 1) arterial hypertension (bBP > 140 mmHg systolic and/or > 90 mmHg diastolic), 2) history or clinical signs of neoplastic disease or disease of the heart, lungs, kidneys or endocrine organs, 3) drug or alcohol abuse (I.e. >14 objects a week for women and 21 for men), 4) medical treatment except oral contraceptives, 5) pregnancy or breast-feeding, 6) significant laboratory abnormalities in the screening test of blood samples (I .e. abnormal haemoglobin, white cell count, plasma sodium, plasma potassium, plasma creatinine, plasma albumin, plasma bilirubin, plasma alanine aminotransferase or serum cholesterol) and urine samples (I.e. albuminuria or glucosuria), 7) abnormal electrocardiogram, 8) blood donation less than one month prior to the study.

In fertile women contraceptive treatment must be used during and after the after the study period (I.e. p-pills, spiral, depot injection of gestagen, sub dermal implantation, hormonal contraceptive vaginal ring and transdermal contraceptive patch).

Withdrawal criteria were development of one or more of the exclusion criteria, bBP increase above 180/105 mmHg during infusion of L-NMMA, withdrawal of consent or lack of compliance.

### Study design

We performed a randomized, crossover double-blinded, placebo- controlled trial. The trial consisted of two treatment periods, placebo or tolvaptan, with an intermediate wash- out period of at least two weeks to eliminate any carryover effects.

### Number of subjects

C_H2O_ was used as the main effect variable. With a minimal relevant difference of 6 ml/min with an estimated standard deviation (SD) of 4 ml/min 18 subjects were needed using a level of significance of 5% and a statistical power of 80%. Due to possible drop outs 20 subjects were included.

### Recruitment

Healthy subjects were recruited by advertising in public institutions and private companies.

### Ethics

This study was approved by the Regional Committee on Health Research Ethics (j. no. M-201223912). The study was conducted in conformity with the principles of the declaration of Helsinki. Written informed consent was obtained from all subjects.

### Effect variables

The primary effect variable was C_H2O._ The secondary effect variables were 1) renal function (GFR, UO, u-AQP2, u-ENaC_γ_, FE_Na_), 2) hemodynamics (bBP, cBP, PWV, AI) and 3) vasoactive hormones (PRC, p-ANG II, p-Aldo, p- AVP).

### Diet

Four days prior to each treatment period all subjects ingested a standardized diet of 11.000 KJ. The diet was composed of 55% carbohydrates, 15% proteins and 30% fat following general dietary guidelines. The sodium content was 150 mmol per day. No additional sodium or other spices was allowed. The daily fluid intake was 2.5 L. No alcohol intake was allowed.

### Experimental procedure

The examinations were conducted in Medical Research from 7:45 AM to 1:00 PM. The procedures were identical on the two examination days. On the morning of each examination day, subjects were given placebo or tolvaptan 15 mg at 6.00 AM. Prior to each examination day a 24-hours urine was collected and a fasting period of eight hours was performed.

An intravenous catheter was placed in each arm to collect blood samples and infuse the ^51^Cr-EDTA. At 8:00 AM and every 30 minutes, an oral water load of 175 ml was given.

BP was measured every 30 minutes from 8:30 AM to 1:00 PM. A bolus infusion of L-NMMA 4.5 mg/kg was given at 11:00 AM, followed by continuous infusion (3 mg/kg/hour) during one hour. The dose was based on results from a dose-finding study made by our team in healthy subjects [9]. During infusion of L-NMMA BP was measured every 5 minutes, and every 15 minutes after infusion of L-NMMA.

Blood samples were drawn every 30 minutes from 8:30 to 1:00 PM and were analyzed for p-Na, p-osm, p-creatinine, p-albumin and p- ^51^Cr-EDTA. Blood samples were drawn for measurement of PRC, p-Aldo, p-Ang II and p-AVP every 60 minutes; at 11:00 AM (baseline), at 12:00 AM (after end of L-NMMA infusion) and at 1:00 PM (60 minutes after end of L-NMMA infusion).

Urine samples were collected by voiding in standing or sitting position every 30 minutes from 9:30 AM to 1:00 PM after collecting BP measurements and blood samples. Otherwise subjects were kept in a supine position in a quiet, temperature-controlled room (22 – 25°C). Baseline period were means of the first three clearance periods. The urine samples were analyzed for osmolality (u-osm), creatinine (u-creatinine), sodium (u-Na), u-AQP2, u-EnaC_γ_ and ^51^Cr-EDTA (u-^51^Cr-EDTA).

Applanation tonometry with SphygmoCor was performed from 10:40 to 11:40 AM.

### Measurements

#### *Renal function*

GFR were measured using the constant infusion clearance technique with 51Cr-EDTA as reference substance.

C_H2O_ was determined using the formula C_H2O_ = UO-C_osm_, where C_osm_ is the osmolar clearance.

Clearance (C) of substance X was calculated as C_X_ = U_X_/(P_X_ x UO), where U_X_ denotes concentration of x in urine, P_X_ denotes concentration of x in plasma, and UO is urine excretion rate.

Fractional exretion of sodium and potassium were determined according to the following formula FE_x_ = (uX * V/pcreatinine/pX* ucreatinine)/GFR, where uX and pX are urine and plasma concentrations respectively, and V is the flow in ml/min during GFR measurement.

#### *Urinary excretion of AQP2 and ENaC_γ_*

Urine samples were kept frozen at -20°C until assayed. U-AQP2 was measured by radioimmunoassay as previously described [20,21]. Antibodies were raised in rabbits to a synthetic peptide corresponding to the 15 COOH-terminal amino acids in human AQP2 to which was added an NH_2_-terminal cystein for conjugation and affinity purification. Minimal detection level was 34 pg/tube/tube. The coefficients of variation were 11.7% (inter-assay) and 5.9% (intra-assay). U-ENaCγ was measured by radioimmunoassay as previously described [22]. Antibodies were raised against the synthetic ENaCγ peptide in rabbits and affinity purified as described previously [23]. Minimal detection level was 48 pg/tube. The coefficients of variation were 14% (inter-assay) and 6.7% (intra-assay). The anti-AQP2 antibody was a gift from Soeren Nielsen, The Water and Salt Research Center, Institute of Anatomy, Aarhus University, Denmark.

#### *Vasoactive hormones in plasma*

Blood samples for measurements of vasoactive hormones were centrifuged for 10 minutes at 2200 G and 4°C. Plasma was separated from blood cells and kept frozen until assayed. PRC was determined using an immunoradiometric assay from CIS Bio International, Gif-Sur-Yvette Cedex, France. Minimal detection level was 1 pg/ml. The coefficients of variation were 0.9-3.6% (intra-assay) and 3.7-5.0% (inter-assay) in the range of 4–263 pg/ml. Aldo was determined by RIA using a kit from Demeditec Diagnostics GmbH, Kiel, Germany. Minimal detection level was 25 pmol/l. The coefficients of variation were 9.0% (inter-assay) and 8.5% (intra-assay). Ang II and AVP were extracted from plasma with C_18_ Sep-Pak (Water associates, Milford, MA, USA), and subsequently determined by radioimmunoassay [24,25]. The antibody against Ang II was obtained from the Department of Clinical Physiology, Glostrup Hospital, Denmark. Minimal detection level was 2 pmol/L. The coefficients of variation were 12% (inter-assay) and 8% (intra-assay). The antibody against AVP was a gift from Professor Jacques Dürr, Miami, Fl, USA. Minimal detection level was 0.2 pmol/L. The coefficients of variation were 13% (inter-assay) and 9% (intra-assay).

#### *Brachial and central blood pressure*

Bbrachial BP pressure was measured using an oscillometer (Omron 705IT) and recorded in accordance with recommendations from the European Society of Hypertension. Brachial systolic and diastolic blood pressure was recorded as the average of duplicate measures. Central BP was measured using applanation tonometry. Recordings of PWA and carotid-femoral PWV were obtained by applanation tonometry (SphygmoCor® CPV system®, AtCor Medical, Sydney, Australia) as double-recordings by a trained observer. Only duplicate recording meeting the quality requirements were included in the final analysis. An operator index of 80 or more was required to accept recordings of a peripheral pulse-wave form. The variation was measured in 10 healthy subjects. The participants rested for 15 minutes in a supine position followed by measurements of brachial blood pressure, PWA and PWV. For intra-observer variation, five measurements were performed by each observer in all ten subjects. For each subject, a coefficient of variation (CV) was calculated for each observer. The mean values of CV’s regarding the ten measurements for each observer were 4-6% for PWV (Observer 1: 6.4%, Observer 2: 3.8%, and Observer 3: 5.6%), and in the same range for PWA (Observer 1: 4.3%, Observer 2: 5.0%, and Observer 3: 5.7%). The inter-observer variation was calculated as means of CV of the average values obtained by the three observers for ten subjects (PWV: 7.5%, PWA: 5.6%).

#### *Routine analyses*

Sodium, albumin, hemoglobin and glucose were measured by routine methods in Department of Clinical Biochemistry, Holstebro Hospital.

#### *Statistics*

Statistical analyses were performed using IBM SPSS statistics version 20.0.0 (SPSS Inc., Chicago, IL, USA). General Linear Model Repeated Measures was used for comparison between and within subjects to test differences between placebo and tolvaptan treatment group at baseline, during and after infusion of L-NMMA. Post-hoc Bonferoni test was used to compare differences within groups at baseline and during L-NMMA infusion, and at baseline and after L-NMMA infusion.

Paired sample *t*-test was performed to compare differences between treatment groups at baseline, during and after infusion of L-NMMA. Statistical significance was at < 0.05 in all analyses. Data with normal distribution are reported as means ± SD. Non-parametric test was performed for data with non-normal distribution, and are reported as medians ± interquartile range or medians with 25^th^ and 75^th^ percentiles.

## Results

### Demographics

We allocated 29 subjects to the study. Ten of the participants were excluded due to lack of compliance (2), difficulties to gain intravenous access (3), arterial hypertension and/or medication (2), withdrawal of consent (3). Nineteen participants completed the study, 12 females and 7 males, with a median age of 25 ± 4 years, weight 72.3 ± 13.4 kg, body mass index 24 ± 3.4 kgm^-2^. p-sodium 141 ± 1.7 mmol/L, p-potassium 3.8 ± 0.3 mmol/L, p-creatinine 77.1 ± 18.7 μmol/L. Systolic brachial blood pressure (SBP) 122 ± 9 mmHg, diastolic brachial blood pressure (DBP) 70 ± 7 mmHg.

### Urine collection before the examination day

Table 1 shows the values of the 24 hours urine collection before each examination day. There were no significant differences in UO, C_H2O_, u-AQP2, u-ENaC_γ_ and u-Na between the two treatment periods.

**Table 1:** **Urine output (UO), free water clearance (C**_**H2O**_**), urinary excretion of AQP2 (u-AQP2), ENaC (u- ENaC**_**γ**_**), sodium (u-Na) and potassium (u-K) during a 24-hours urine collection before each examination day in a randomized, placebo-controlled, double-blind, crossover study of 19 healthy subjects**

	**Before each examination day**		**p (paired** ***t*-test)**
	**Placebo**	**Tolvaptan**	
**UO (ml/24 h)**	1969 ± 448	2025 ± 514	0.51
**C**_**H2O**_**(ml/min)**	-0.46 ± 0.61	-0.47 ± 0.57	0.99
**U-AQP2 (ng/min)**	1.10 ± 0.32	1.12 ± 0.27	0.57
**U-ENaCγ (pg/min)**	716 ± 374	705 ± 376	0.87
**U-Na (mmol/24 h)**	103 ± 11	101 ± 8	0.652
**U-K (mmol/24 h)**	55 ± 19	51 ± 14	0.472

Data are shown as means with ± SD. Paired *t*-test was used for comparison between groups.

### GFR

**Figure 1:**
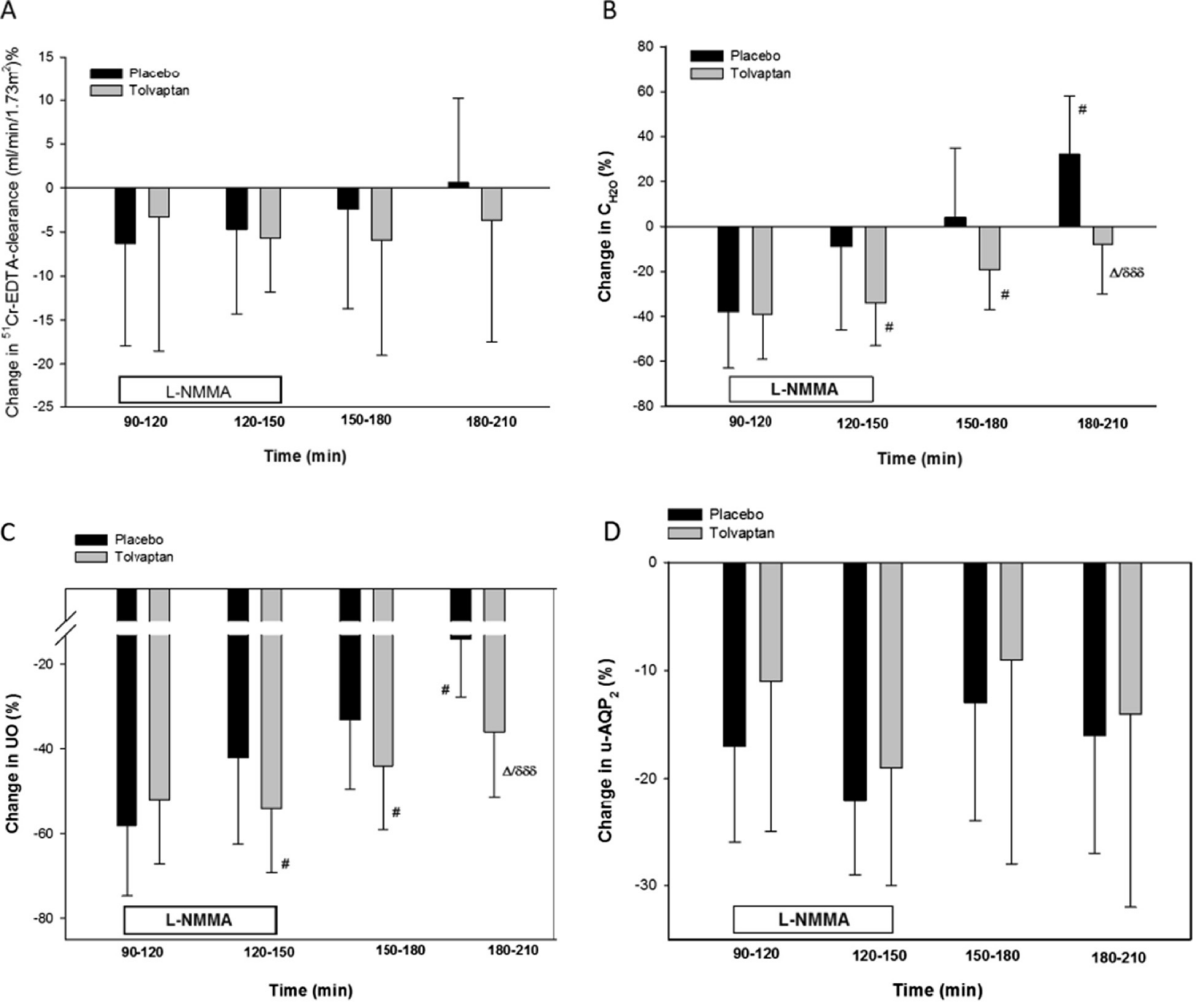
**Relative changes in GFR (A), C**_**H2O**_**(B), UO (C) and u-AQP**_**2**_**(D) during NO-inhibition.** Values are mean ± SEM. General linear model (GLM) with repeated measurements was performed for comparison within the group (δ) and interventions as between (∆) subject factor. Paired *t*-test or Wilcoxon signed rank test (#) was performed for comparison within treatment group at baseline vs during infusion period 90–150 min, and baseline vs post infusion 150–210 min. #/∆ p < 0.05; δδδ p < 0.0001.

At baseline, GFR was the same after both treatments (Table 2). In response to L-NMMA infusion, GFR was significantly reduced during both treatments, but no differences were found between treatments. The relative decreases in GFR were similar after placebo and tolvaptan (Figure 1).

**Table 2:** **Effect of tolvaptan at baseline and during inhibition of the nitric oxide system on GFR (51-CrEDTA-clearance), urinary output (OU), free water clearance (C**_**H2O**_**), urinary aquaporin2 excretion rate (u-AQP2), urinary ENaC**_**γ**_**excretion rate (u-ENaC**_**γ**_**) and fractional excretion of sodium (FE**_**Na**_**) in a randomized, double-blind, placebo-controlled, crossover study of 19 healthy subjects**

**Periods**	**Baseline**	**L-NMMA**		**Post infusion**		**P (GLM-within)**
		**90-120 min**	**120-150 min**	**150-180 min**	**180-210 min**	
^**51**^**Cr-EDTA-clearance (ml/min/1.73 m2)**						
Placebo	99 ± 5	93 ± 10^***^	92 ± 8^***^	94 ± 8^***^	97 ± 9	0.522
Tolvaptan	97 ± 9	91 ± 12^***^	93 ± 9^***^	90 ± 12^***^	94 ± 12	
p (GLM between) 0.527						
p (paired *t*-test, between)	0.403	0.533	0.583	0.118	0.197	
**UO (ml/min)**						
Placebo	7.4 ± 1.3	3.0 ± 1.2^***^	4.2 ± 1.3^***^	4.9 ± 1.2^***^	6.3 ± 1.1	<0.0001
Tolvaptan	8.9 ± 1.7	4.3 ± 1.8^***^	4.2 ± 1.8^***^	4.9 ± 1.3^***^	5.7 ± 1.6	
p (GLM between) 0.230						
p (paired *t*-test, between)	0.009	0.026	0.965	0.861	0.124	
**C**_**H2O**_**(ml/min)**						
Placebo	4.6 ± 1.1	2.9 ± 1.0^***^	3.9 ± 1.2^***^	4.6 ± 1.0^***^	5.9 ± 1.0	<0.0001
Tolvaptan	5.8 ± 1.1	4.4 ± 1.8^*^	4.0 ± 1.6^*^	3.9 ± 1.2	5.2 ± 1.4	
p(GLM between) 0.185						
p (paired *t*-test, between)	0.002	0.009	0.756	0.063	0.097	
**u-AQP2 (ng/min)**						
Placebo	1.30 ± 0.27	1.04 ± 0.36^*^	0.99 ± 0.19^***^	1.06 ± 0.24^***^	1.11 ± 0.20	0.444
Tolvaptan	1.32 ± 0.28	1.08 ± 0.39	1.04 ± 0.23^***^	1.17 ± 0.32^***^	1.10 ± 0.21	
p (GLM between) 0.614						
p (paired *t*-test, between)	0.771	0.427	0.382	0.044	0.665	
**u-ENaC**_**γ**_**(pg/min)**						
Placebo	476	-	367	-	442^**^	
	(398; 640)		(296; 756)		(308; 605)	
Tolvaptan	320	-	430 ^***^	-	567^***^	
	(281; 379)		(361; 492)		(322; 537)	
p (Wilcoxon’s signed rank test, between)	<0.001		0.355		0.227	
**FE**_**Na**_						
Placebo	1.17 ± 0.62	0.77 ± 0.35^*^	0.78 ± 0.37^*^	1.12 ± 0.41	1.07 ± 0.35	0.945
Tolvaptan	1.29 ± 0.45	0.89 ± 0.34^*^	0.87 ± 0.32^*^	1.22 ± 0.30	1.18 ± 0.30	
p (GLM between) 0.328						
p (paired *t*-test, between)	0.326	0.073	0.073	0.198	0.200	

Data are shown as mean with ± SD or medians with 25^th^ and 75^th^ percentiles in parentheses. General linear model (GLM) with repeated measures was performed for comparison within the group and intervention as between subject factor. Post-hoc Bonferoni test (*) was used for comparison of infusion period 90–150 min vs baseline and post infusion period 150–210 vs baseline. Paired *t*-test or Wilcoxon signed rank test was used for comparison between treatment group at baseline and during infusion period 90–150 min, and at baseline and post infusion period 150 – 210 min. ^*^p < 0.05; ^**^p < 0.001;^***^p < 0.0001.

### Tubular handling of water and sodium

Absolute values of UO and C_H2O_ after treatment with placebo and tolvaptan are shown in Table 2. The relative changes after L-NMMA infusions are shown in Figure 1. At baseline, UO and C_H2O_ were significantly higher during tolvaptan treatment compared with placebo (p = 0.009 and p = 0.002, respectively). During L-NMMA infusion, UO and C_H2O_ were significantly decreased after both treatments. However, UO and C_H2O_ were approximately 30% lower in the first 30 minutes (Period: 90–120 min) during L-NMMA infusion in the placebo group compared to the tolvaptan group (p = 0.026 and 0.009 respectively). During the following 30 minutes (period: 120–150 min.), UO and C_H2O_ were similar. The relative decrease in C_H2O_ and UO were significant within both treatments, but the relative decrease in UO and C_H2O_ were significantly more pronounced in the tolvaptan group (p = 0.018 and p = 0.008 respectively).

At baseline, Table 2 shows that FE_Na_ was similar during both treatments. During L-NMMA infusion, FE_Na_ was significantly decreased and to the same extent during both treatments.

### U-AQP2 and U-ENaC

During baseline conditions, u-AQP2 was the same after tolvaptan and placebo (Table 2). In response to L-NMMA infusion, u-AQP2 decreased significantly and to the same extent after both treatments. The relative changes in u-AQP2 were similar during and after L-NMMA infusion in the treatment groups (Figure 1).

**Figure 2:**
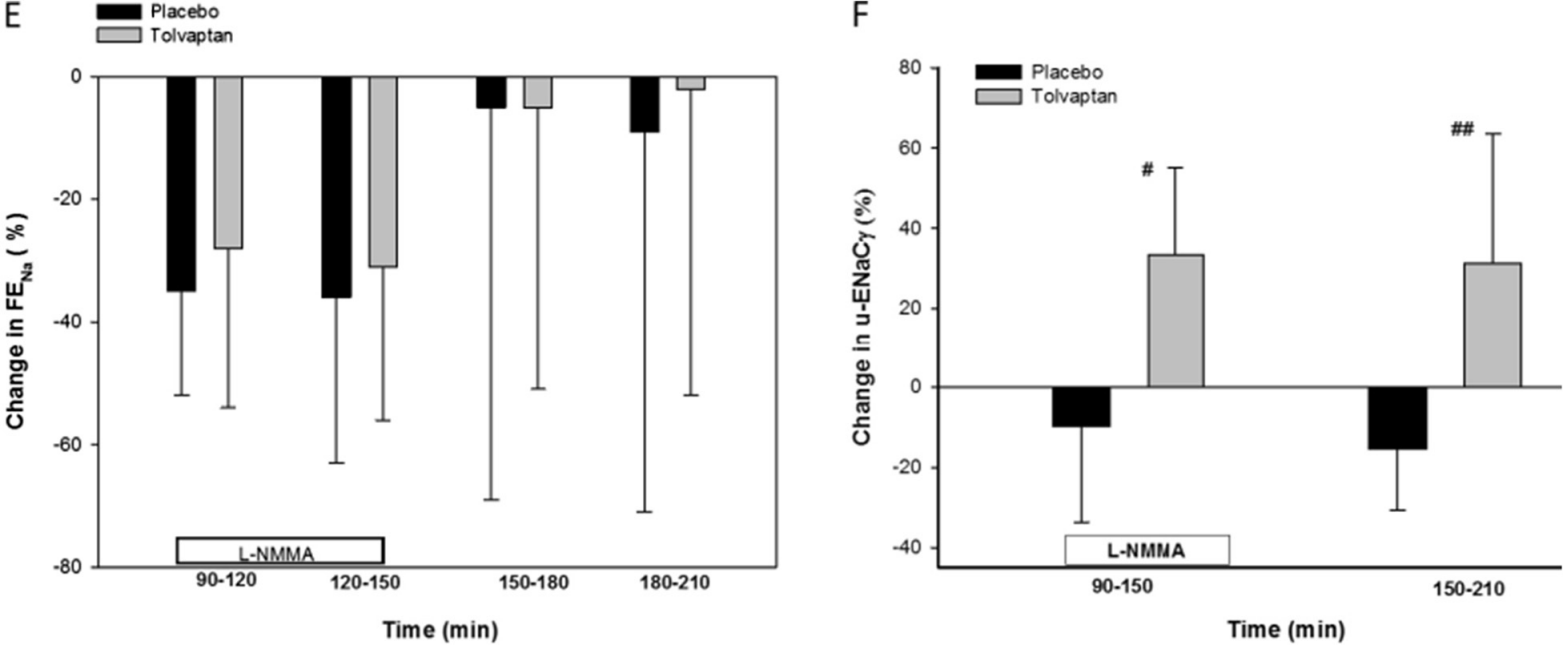
**Relative changes in FE**_**Na**_**(E) and u-ENaCγ (F) during inhibition of the NO system.** Values are mean ± SEM. General linear model (GLM) with repeated measurements was performed for comparison within the group (δ) and interventions as between (∆) subject factor. Paired *t*-test or Wilcoxon signed rank test (#) was performed for comparison within treatment group at baseline vs during infusion period 90–150 min, and baseline vs post infusion 150–210 min. # p < 0.05; ## p < 0.001.

U-ENaC_γ_ was approximately 33% lower during tolvaptan treatment compared to placebo at baseline (p = 0.002). During L-NMMA infusion, u-ENaC_γ_ was reduced by L-NMMA infusion after placebo, whereas a significant increase was measured after tolvaptan (p < 0.001). U-ENaC_γ_ decreased 9.76 ± 23.94% after placebo and increased 33.40 ± 21.86% after tolvaptan in the L-NMMA infusion period (Figure 2).

### Plasma sodium, plasma osmolarity and plasma potassium

During baseline conditions, p-Na, p-Osm and p-K were significantly higher in the tolvaptan group compared to the placebo group (Table 3). In response to L-NMMA infusion, p-Na and p-Osm remained significantly higher duing tolvaptan treatment compared with placebo, whereas no differences were measured in p-K between the treatments.

**Table 3:** Effect of tolvaptan at baseline and during inhibition of the nitric oxide system on plasma concentration of sodium and potassium and plasma osmolality in a randomized, placebo-controlled, double-blind, crossover study of 19 healthy subjects

**Periods**	**Baseline**	**L-NMMA**		**Post infusion**		**p (GLM-within)**
	**0-90 min**	**90-120 min**	**120-150 min**	**150-180 min**	**180-210 min**	
**p-sodium (mmol/l)**						
Placebo	140 ± 2	139 ± 1	139 ± 2^**^	139. ± 2	138 ± 2^**^	0.272
Tolvaptan	143 ± 2	143 ± 2	141 ± 3^**^	141 ± 2	141 ± 2^*^	
P (Wilcoxon’s signed rank test)	0.001	0.001	0.002	0.001	0.001	
**p- osm (mosm/kg)**						
Placebo	285 ± 3	285 ± 4	283 ± 4^**^	284 ± 4^**^	283 ± 4^***^	0.352
Tolvaptan	291 ± 3	291 ± 3	291 ± 3	291 ± 3	290 ± 3	
p (GLM between) <0.0001						
p (paired *t*-test)	<0.001	<0.001	<0.001	<0.001	<0.001	
**p-potassium (mmol/l)**						
Placebo	3.8 ± 0.2	3.8 ± 0.3	3.9 ± 0.3	3.9 ± 0.2	3.8 ± 0.1	0.325
Tolvaptan	3.9 ± 0.2	3.9 ± 0.2	4.0 ± 0.2	4.0 ± 0.1	4.0 ± 0.1	
p (Wilcoxon’s signed rank test)	0.028	0.117	0.272	0.929	0.430	

Data are shown as mean with ± SD or medians with ± interquartile range. General linear model (GLM) with repeated measures was performed for comparison within the group and intervention as between subjects factor. Post-hoc Bonferoni test (*) was used for comparison of infusion period 90–150 min vs baseline and post infusion period 150–210 vs baseline. Paired *t*-test or Wilcoxon signed rank test was used for comparison between treatment group at baseline vs during infusion period 90–150 min, and at baseline vs post infusion period 150 – 210 min. ^*^p < 0.05; ^**^p < 0.001;^***^p < 0.0001.

### Vasoactive hormones

At baseline, PRC, p-AngII and P-Aldo were the same after tolvaptan and placebo (Table 4). PRC was significantly decreased during inhibition of NO synthesis in both treatment groups (p < 0.001), but no difference was observed between groups. P-AngII fell significantly only in the tolvaptan group (p = 0.001), but tended to fall also during placebo. P-Aldo was unchanged during both treatments.

**Table 4:** Effect of tolvaptan at baseline and during inhibition of the nitric oxide system on plasma concentrations of renin(PRC), angiotensin II (P-AngII), aldosterone (P-aldo) and vasopressin (P-AVP) in a randomized, placebo-controlled, double-blind, crossover study of 19 healthy subjects

**Periods**	**Baseline**	**L-NMMA**	**Post infusion**	**P (GLM-within)**
	**11:00 AM**	**12:00 AM**	**1:00 PM**	
**PRC(pg/ml)**				
Placebo	8.1 ± 4.3	6.1 ± 3.2^**^	5.9 ± 3.2^**^	0.670
Tolvaptan	9.9 ± 6.8	7.5 ± 4.6^**^	7.5 ± 5.4^**^	
p (GLM between) 0.305				
p (paired *t*-test, between)	0.101	0.050	0.038	
**P-AngII (pg/ml)**				
Placebo	9.5 ± 4	8.6 ± 3.7	8.0 ± 3.3^*^	0.156
Tolvaptan	11.9 ± 6.2	9.4 ± 4.6^*^	9.5 ± 5.1^*^	
p (GLM between) 0.686				
p (paired *t*-test, between)	0.094	0.403	0.117	
**P- Aldo (pmol/L)**				
Placebo	70 ± 2	75 ± 2	64 ± 2	0.949
Tolvaptan	71 ± 2	78 ± 2	66 ± 1	
p (GLM between) 0.899				
p (paired *t*-test, between)	0.962	0.785	0.840	
**P- AVP (pg/ml)**				
Placebo	0.20 ± 0.15	0.20 ± 0.15	0.20 ± 0.20	
Tolvaptan	0.70 ± 0.45	0.70 ± 0.55	0.70 ± 0.60	
p (Wilcoxon’s signed rank test, between)	<0.001	<0.001	<0.001	

Data are shown as mean with ± SD or medians with ± interquartile range. General linear model (GLM) with repeated measures was performed for comparison within the group and intervention as between subject factor. Post-hoc Bonferoni test (*) was used for comparison of infusion period 90–150 min vs baseline and post infusion period 150–210 vs baseline. Paired *t*-test or Wilcoxon signed rank test was used for comparison between treatment group at baseline vs during infusion period 90–150 minu, and at baseline vs post infusion period 150 – 210 min. ^*^p < 0.05; ^**^p < 0.001;^***^p < 0.0001.

A highly significant and sustained 3–fold increase in p-AVP was measured during treatment with tolvaptan compared to placebo (Placebo: 0.20 ± 0.15 vs. 0.70 ± 0.45 pg/ml, p <0.0001). P-AVP did not change from baseline levels during L-NMMA infusion.

### Effects of L-NMMA on brachial bloodpressure

**Figure 3:**
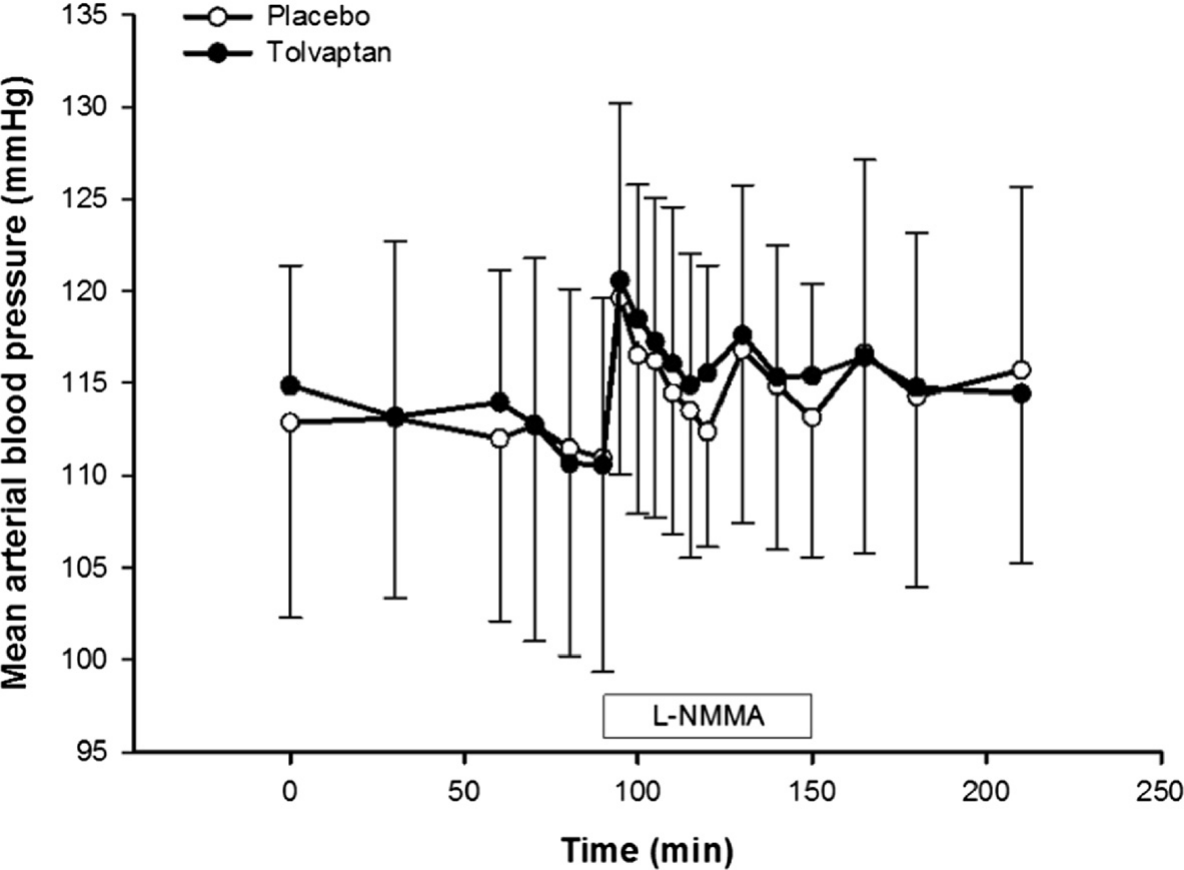
**Effect of tolvaptan on bBP during inhibition of the NO system.** Values are mean ± SEM. Baseline brachial blood pressure (bBP) was defined as a mean of the four measurements 30 min prior to L-NMMA infusion. A stable bBP was achieved for the last 40 min of L-NMMA infusion. A mean of the six measurements from last 40 minutes of L-NMMA infusion was used to calculate changes from baseline. Post-hoc Bonferoni test was used for comparison of the mean of the last 40 min of infusion vs. baseline (p = 0.011 in placebo vs p < 0.0001 in tolvaptan). Paired *t*-test was performed for comparison of the last 40 min of infusion vs. baseline between treatment groups.

Within the first five minutes of L-NMMA bolus infusion, BP peaked in both groups and then gradually declined over the first 20 min of infusion (Figure 3). During the remaining 40 minutes of infusion, BP changes were similar in both groups (p = 0.221 for SBP and p = 0.678 for DBP with GLM). An average of the six measurements from the last 40 minutes of L-NMMA infusion was compared to baseline blood pressure. L-NMMA caused a significant increase in SBP (4 ± 3 mmHg in placebo vs. 3 ± 2 mmHg in tolvaptan) and bDBP (7 ± 6 mmHg in placebo vs. 6 ± 7 mmHg in tolvaptan). No significant differences were found between treatments (p = 0.374 for SBP and p = 0.606 for DBP).

### Effects of tolvaptan on brachial blood pressure

At baseline, SBP and DBP did not differ between treatments, whereas pulse rate was significantly higher in the placebo group (Table 5). L-NMMA infusion induced a significant increase in both SBP and DBP during both treatments, but differences were measured between treatments. During L-NMMA infusion, pulse rate fell similarly in both groups.

**Table 5:** Effect of tolvaptan at baseline and during inhibition of the nitric oxide system on brachial systolic- and diastolic blood pressure (SBP and DBP), and pulse rate in a randomized, placebo-controlled, double-blind, crossover study of 19 healthy subjects

**Periods**	**Baseline**	**L-NMMA**		**Post infusion**		**p (GLM-within)**
	**0-90 min**	**90-120 min**	**120-150 min**	**150-180 min**	**180-210 min**	
**SBP (mmHG)**						
Placebo	112 ± 8	113 ± 9	115 ± 8^*^	115 ± 9^*^	116 ± 10^*^	0.170
Tolvaptan	112 ± 11	115 ± 9^*^	116 ± 9^*^	116 ± 10^*^	114 ± 9	
p (GLM between) 0.876						
p (paired *t*-test, between)	0.833	0.117	0.272	0.929	0.430	
**DBP (mmHg)**						
Placebo	61 ± 5	68 ± 6^***^	67 ± 6^***^	67 ± 5^*^	65 ± 7	0.688
Tolvaptan	63 ± 5	69 ± 7^***^	68 ± 6^***^	67 ± 6	66 ± 7	
p(GLM between) 0.606						
p (paired *t*-test, between)	0.049	0.467	0.259	0.907	0.454	
**Pulse rate (BPM)**						
Placebo	57 ± 10	52 ± 9^***^	53 ± 9^***^	55. ± 11^***^	58 ± 12^***^	0.889
Tolvaptan	55 ± 10	50 ± 9^***^	51 ± 9^***^	53 ± 10^***^	56 ± 11^*^	
p (GLM between) 0.527						
p (paired *t*-test, between)	0.016	0.022	0.007	0.013	0.003	

Data are shown as mean with ± SD or medians with ± interquartile range. General linear model (GLM) with repeated measures was performed for comparison within the group and intervention as between subjects factor. Post-hoc Bonferoni test (*) was used for comparison of infusion period 90–150 min vs baseline and post infusion period 150–210 vs baseline. Paired *t*-test or Wilcoxon signed rank test was used for comparison between treatment group at baseline vs during infusion period 90–150 min, and at baseline vs post infusion period 150–210 min. ^*^p < 0.05; ^**^p < 0.001;^***^ p < 0.0001.

### Central blood pressure

At baseline, no difference was observed in PWV, cBP and AI between tolvaptan and placebo (Table 6). During L-NMMA infusion, PWV increased significantly in the tolvaptan group (p = 0.012), whereas it remained unchanged in the placebo group. Systolic cBP and AI increased in the same way in the tolvaptan group, but remained unchanged in the placebo group. The changes were similar between groups. During l-NMMA infusion diastolic cBP increased significantly after both treatments.

**Table 6:** Effect of tolvaptan at baseline and during inhibition of the nitric oxide system on pulse wave velocity (PWV), augmentation index (AI), central diastolic and systolic blood pressure (CBDP and CSBP) in a randomized, placebo-controlled, double-blind, crossover study of 19 healthy subjects

**Periods**	**Baseline**	**L-NMMA**
**PWV(m/s)**		
Placebo	5.3 ± 0.7	5.5 ± 0.5
Tolvaptan	5.3 ± 0.6	5.6 ± 0.7^*^
p (paired *t*-test, between)	0.652	0.929
**AI**		
Placebo	0.0 ± 18.7	3.7 ± 18.7
Tolvaptan	1.2 ± 18.3	7.2 ± 17.5^*^
p (paired *t*-test, between)	0.646	0.064
**CSBP**		
Placebo	100 ± 7	105 ± 4
Tolvaptan	100 ± 4	107 ± 7^*^
p (paired *t*-test, between)	0.871	0.351
**CDBP**		
Placebo	66 ± 12	70 ± 8^*^
Tolvaptan	64 ± 5	68 ± 8^*^
p (Wilcoxon signed rank test, between)	0.440	0.622

Data are shown as mean with ± SD or medians with ± interquartile range. Paired *t*-test or Wilcoxon signed rank test was used for comparison within (*) treatment group at baseline vs during infusion period 90–150 min, and at baseline vs post infusion period 150 – 210 min. ^*^p < 0.05.

## Discussion

In the present study, we examined the effect of short-term treatment with tolvaptan on renal tubular function, vasoactive hormones and central hemodynamics, during basal conditions and during inhibition of the NO-system with L-NMMA in healthy subjects. During baseline conditions, tolvaptan increased UO and C_H2O._ An expected decrease in u-AQP2 was not measured and counteracted by a threefold increase in p-AVP. Tolvaptan did not change FE_Na_ and decreased u-ENaCγ as expected. During NO-inhibition, UO and C_H2O_ decreased after both treatments, but the decrease after tolvaptan was significantly more pronounced than after placebo. In contrast, FE_Na_ was decreased similarly after both tolvaptan and placebo during NO-inhibition. U-AQP2 decreased to the same extent after both treatments, whereas u-ENaCγ decreased after tolvaptan and increased after placebo. The present study is the first randomized, double-blinded, placebo-controlled crossover trial to measure the effect of tolvaptan on renal tubular function, vasoactive hormones and central hemodynamics during inhibition of the NO-system with L-NMMA. The dose of L-NMMA was based on a previous dose–response study [9]. We demonstrated an infusion method in which a steady NOS inhibition was obtained, and in a dose dependent manner L-MNNA infusion increased blood pressure.

### Renal handling of water

During baseline conditions, UO and C_H2O_ increased after tolvaptan as expected. However, after NO- inhibition, the decrease in UO and C_H2O_ was significantly more pronounced after tolvaptan than placebo, but p-AVP was unchanged. Thus, tolvaptan seemed to potentiate the reduction in UO and C_H2O_ induced by L-NMMA. This could be explained by interference of NO with renal water excretion in the principal cells in the distal part of the nephron by a partly AVP-dependent mechanism, resulting in an increase in renal water excretion. This is a new and original observation. However, we cannot exclude an impact of NO on water excretion through an AVP-dependent mechanism, too.

In the distal part of the nephron, water is absorbed via AQP2 [1]. AVP exhibits a short-term effect through the cAMP pathway to regulate translocation of preformed AQP2 proteins from cytosolic vesicles to the apical plasma membrane of the collecting duct principal cells, thereby increasing water permeability across the collecting duct [3]. In addition, AVP exhibits a long-term effect through its action on the cAMP-responsive element in the AQP2 promoter site, thereby enhancing the AQP2 gene expression. This long-term regulation determines the abundance of AQP2 water channels available for the modulation of the apical membrane's water permeability [1,26,27]. This is in agreement with results in several experimental and clinical studies, which have demonstrated that treatment with vasopressin or 1-desamino-8-D-arginine-vasopressin (dDAVP), a V2R agonist, enhanced translocation of AQP2 water channels to the apical membrane and results in water retention [2]. Surprisingly, during baseline conditions, u-AQP2 was the same after tolvaptan and placebo. However, the lack of decrease in u-AQP2 after tolvaptan can be attributed to the high level of p-AVP, which counteracts the effect of tolvaptan on the V2 receptor. Another explanation might be that the baseline period was too short to reflect a decrease in u-AQP2 after tolvaptan. During NO inhibition, u-AQP2 was suppressed to the same extent both after tolvaptan and placebo. Conflicting results have been reported regarding the effect of NO on AQP2 water channels. Some results suggested an effect via a cGMP dependent pathway, and others via an AVP dependent mechanism [28-30]. We demonstrated that u-AQP2 fell in response to NO inhibition during both treatments. From the placebo treatment, our results strongly support the assumption that L-NMMA decreased translocation of AQP2 channels to the apical membrane, and thereby reduced water absorption via reduced water transport via the AQP2 water channels. Tolvaptan treatment, however, potentiated the effect of L-NMMA on UO and C_H2O,_ and thereby increased the absorption of water significantly. This means that the increase in renal water excretion induced by NO seems to be at least partly mediated by an AVP-dependent mechanism. During NO-inhibition, the lack of differences in uAQP2 between tolvaptan and placebo can be attributed to the fact that u-AQP2 was suppressed to a low level during both treatments by L-NMMA, and a difference between the two treatments could not be detected during this condition.

### Renal handling of sodium

During baseline conditions, FE_Na_ was the same after tolvaptan and placebo. L-NMMA reduced FE_Na_ to the same extent after both treatments. The role of AVP in renal sodium handling is debated. Activation of V2R by AVP increased water permeability of the luminal membrane through its action on AQP2, but also increased sodium absorption by of ENaC [2,5]. According to studies in rats, the effect of AVP on ENaC depended on the plasma concentration of AVP [2]. Within the physiologic range, AVP promoted antinatriuresis in the distal nephron, mediated by V2R. In healthy subjects, Blanchard et al. [31] showed that the antinatriuretic effect of dDAVP was amiloride sensitive, and thus related to vasopressin’s stimulatory effect on sodium absorption to be mediated by ENaC. We found a lower u-ENaCγ during tolvaptan treatment during baseline conditions, which is in agreement with a blockade of ENaC by VR2 antagonism. Our findings are in agreement with previous clinical studies of the effect of V2R agonism on sodium excretion by ENaC in patients with nephrogenic diabetes insipidus (NDI). Administration of dDAVP induced an antinatriuretic effect in NDI patients with an intact V2R, but not in those with defective V2R [32]. This is also in accordance with studies in rats, where a natriuretic effect was measured after V2R antagonism, but the aquaretic effect exceeded the natriuretic effect [2,33]. During NO-inhibition, u-ENaCγ decreased after placebo and increased after tolvaptan. Thus, NO-inhibition antagonized the effect of tolvaptan on ENaC. Although the variation in u-ENaC_γ_ was considerable, this suggests that NO interferes with the transport via ENaC with an AVP-dependent mechanism, supporting the assumption that the AVP-cAMP pathway of ENaC regulation is relevant for sodium homeostasis in humans [28,32]. However, the decrease in sodium reabsorption after L-NMMA was not mediated by a reduced transport via ENaC, but must be due to L-NMMA induced change in tubular sodium absorption at another location in the nephron.

### Vasoactive hormones

We found no differences in PRC, p-Aldo and p-AngII at baseline in the two treatments, whereas p-AVP increased approximately 3-fold in response to treatment with tolvaptan. Most likely, this rise in endogenous p-AVP is a compensatory release of the hormone in response to the rise in p-osmolality induced by V2R antagonism [34]. During L-NMMA infusion, PRC fell after placebo and tolvaptan treatment, whereas p-ANG.II tended to fall after both treatment, but only significantly in the tolvaptan group. This is in good agreement with previous results from our group [9]. P-AVP remained unchanged during L-NMMA infusion in both treatment groups. Thus, NO does not seem to have a regulatory effect on the release of AVP.

### Central and brachial blood pressure

We observed no changes in bBP and cBP during treatment with tolvaptan despite of the increase in p-AVP in the tolvaptan group. The effect of AVP on BP is mediated by its action on V1a receptors. Activation of V1a receptor increases BP by a direct effect on vascular smooth muscle cells, by which AVP stimulates vasoconstriction and increases BP [2]. It is well documented that treatment with V2 receptor antagonists causes compensatory increase in the plasma concentration of vasopressin, which stimulates the V1a receptors in the vascular smooth muscle cells and thus affect central and peripheral hemodynamics [2]. Our study is the first to measure both bBP and cBP during V2R antagonism before, during and after inhibition of systemic NO synthesis in healthy subjects. Applanation tonometry was performed under standardized conditions. The method is described and evaluated elsewhere [35]. We showed that a 3-fold increase in AVP was not sufficient to increase BP via an activation of V1aR, which is consistent with previous studies [2]. However, pulse rate values were approximately 2 beats per minute lower in the tolvaptan group compared to the placebo group. Most likely, this slight decrease in pulse rate is due to increased baroreceptor sensitivity exerted by AVP [36,37]. However this had no net effect on BP. L-NMMA infusion caused an increase in bBP and cBP to the same extent during both treatments. The results are in agreement with the results recently reported by our group [9] on the effect on systemic NO inhibition on BP. Thus, in the given dose of tolvaptan, no effect is measured on bBP and cBP.

### Plasma levels of sodium, potassium and osmolality

We found significantly higher p-Na, p-K and p-osmolality in baseline during treatment with tolvaptan. The response is in agreement with previous clinical studies [12,17,33]. The SALT [17] investigators have demonstrated that serum sodium concentration was significantly higher within 8 hours after administration of tolvaptan 15 mg compared to placebo. It should be noticed that their study was conducted in patients with hypervolemic and euvolemic hyponatremia. In response to NO inhibition, p-osm and p-K did not change. In contrast, p-Na decreased in both treatment groups in the last 30 minutes of L-NMMA infusion. It is easily explained by the decrease of UO, C_H2O_ and u-Na. Recently, our group reported similar results during L-NMMA infusion in healthy subjects [9].

### Strengths and limitations

The major strength of the present study was the design as a randomized, double-blinded, placebo-controlled crossover trial in healthy subjects. Diet, sodium and fluid intake were predefined and controlled during the study to avoid confounding of the results. It is a weakness of the study that we did not measure total plasma or urine nitrite and nitrate as indices of NO synthesis to ensure abrogated systemic NO production. However, during L-NMMA infusion we measured an increase in mean arterial blood pressure and a decrease in GFR, UO, C_H2O_, u-AQP2 and FE_Na_ which clearly indicated NO inhibition.

## Conclusion

During baseline conditions, tolvaptan increased renal water excretion. During NO-inhibition, the more pronounced reduction in renal water excretion after tolvaptan than after placebo indicates that NO promotes renal water excretion in the principal cells by at least a partly an AVP-dependent mechanism. The lack of decrease in u-AQP2 by tolvaptan could be explained by a counteracting effect of a three-fold increase in plasma vasopressin. The antagonizing effect of NO-inhibition on u-ENaC suggests that NO interferes with the transport via ENaC by an AVP-dependent mechanism.

## Competing interests

The authors declare that they have no competing interests.

## Authors’ contributions

SAT participated in the coordination of the study, recruitment of subjects, carried out the experiments, performed statistical analysis, interpretation of data and drafted the manuscript. FHM assisted in the experimental part and helped to draft the manuscript. JMJ assisted in the experimental part and helped to draft the manuscript. JNB helped to draft the manuscript. EBP conceived of the study, participated in the design of the study, was responsible for the laboratory analyses and helped to draft the article. All authors read and approved the final manuscript.

## Pre-publication history

The pre-publication history for this paper can be accessed here:

http://www.biomedcentral.com/1471-2369/15/100/prepub
